# Determination of the Minimum Uncut Chip Thickness of Ti-6Al-4V Titanium Alloy Based on Dead Metal Zone

**DOI:** 10.3390/mi15121458

**Published:** 2024-11-29

**Authors:** Yaohui Zheng, Wentao Huang, Yangyang Liu, Pengchao Duan, Yingxiao Wang

**Affiliations:** 1 School of Engineering Training Center, Shenyang Aerospace University, Shenyang 110136, China; zhengyh214@sau.edu.cn; 2 Key Laboratory of Rapid Development & Manufacturing Technology for Aircraft, Shenyang Aerospace University, Ministry of Education, Shenyang 110136, China; duanpengchao@stu.sau.edu.cn (P.D.); wangyingxiao@stu.sau.edu.cn (Y.W.); 3 School of Mechatronics Engineering, Shenyang Aerospace University, Shenyang 110136, China

**Keywords:** micro turning, minimum uncut chip thickness, dead metal zone, finite element method

## Abstract

In Ti-6Al-4V titanium alloy micro-machining, since the uncut chip thickness (UCT) is comparable to the radius of the tool cutting edge, there exists a minimum uncut chip thickness (MUCT), and when the UCT is smaller than the MUCT, the plowing effect dominates the cutting process, which seriously affects the machined surface quality and tool life. Therefore, the reliable prediction of the MUCT is of great significance. This paper used Deform to establish an orthogonal cutting simulation model, studied the effect of the dead metal zone (DMZ) on the micro-cutting material flow, determined the DMZ range, and proposed a new method for determining the MUCT based on the DMZ. Cutting experiments were conducted to verify the accuracy of the simulation model firstly by cutting force, and then confirming the accuracy of the DMZ-based MUCT determination method through chip analysis and surface quality analysis. Finally, the effects of different cutting conditions on DMZ and MUCT were further investigated using the proposed DMZ-based MUCT determination method. The results show that the MUCT of Ti-6Al-4V titanium alloy is 4.833 μm for a tool cutting edge radius of 40 μm and a cutting speed of 10 m/min. The DMZ boundary can be used as the boundary of micro-cutting plastic deformation, and the ratio of MUCT to cutting edge radius, *h_p_*/*r_n_* will gradually decrease with the increase in the tool cutting edge radius and the cutting speed.

## 1. Introduction

In recent years, Ti-6Al-4V titanium alloy has been widely developed and utilized in aerospace, automotive industry, biomedical, and other fields due to its excellent mechanical properties, good corrosion resistance, and biocompatibility [1]. However, due to the characteristics of high strength, low thermal conductivity, and easy work hardening, Ti-6Al-4V often faces a lot of difficulties in machining, among which tool wear, low machining efficiency, and poor surface quality are particularly prominent [2]. As a precision machining method, micro turning can realize complex structure machining with high accuracy and quality [3], which can be used to manufacture precision parts. Differing from the conventional cutting process, the tool cutting edge radius in micro turning is comparable to the size of the uncut chip thickness (UCT), and there is an obvious plow effect and negative rake angle effect in the cutting process, and there is a minimum uncut chip thickness (MUCT); when the UCT is smaller than the MUCT, the material flowing to the cutting edge will not be converted into chips, but will be plowed out from under the tool fillet [4]. MUCT, as the main factor affecting the formation of micro-machining chips, influences the machining accuracy and quality of micro-machining [5], so the reliable evaluation of MUCT is crucial for micro-machining.

Currently, there is still no uniform standard for MUCT calculation and evaluation methods, and the research methods mainly include analytical methods, finite element methods, and experimental methods. Most analytical methods are based on determining the stagnation point on the rounded edge of the cutting tool to determine the MUCT. Storch and Zawada-Tomkiewicz [6] developed an analytical model of orthogonal cutting MUCT based on the tangential cutting force at the stagnation point being zero. Son et al. [7] used the Merchant cutting force model and the cutting force balance at the stagnation point to develop a MUCT mathematical model, and the model of the MUCT is only related to the tool cutting edge radius and the friction angle. Przestacki et al. [8] established a MUCT model for angled cutting by assuming that the cutting force component and its increment are located at the center of gravity of the chip cross-section. Wojciechowski [9] established a MUCT model for the micro-milling process and found that the MUCT increases with the increase in the cutting speed. The role of heat of cutting in the process of chip generation was confirmed and the MUCT value of 0.17~0.29*r_n_* (tool cutting edge radius) was obtained. Wu et al. [10] established a model for calculating the effective fillet angle of the micro-milling process based on the averaging method and established a method for calculating the MUCT based on the effective rake angle, which resulted in the MUCT of 0.17*r_n_* for copper.

The scale of micro turning is very small, and it is difficult to study the cutting process and chip generation, but with the continuous innovation of numerical simulation technology, many scholars have carried out a lot of research on the MUCT and micro turning process through the establishment of simulation models. Guo et al. [11] established a simulation model of the micro turning of oxygen-free copper by using the SPH method, and simulation experiments were carried out with different *h*/*r_n_* (the ratio of the UCT to the tool cutting edge radius), and the results showed that when *h*/*r_n_* is less than or equal to 0.5, the shear effect is strongly suppressed, and the workpiece material mainly flows to the bottom of the tool fillet and forms the machined surface. Zhang et al. [12] established a finite element model of the cutting of Al 7075-T6 aluminum alloy by a cemented carbide tool, and determined the stagnation point and the MUCT by the node displacement speed, which resulted in the MUCT of about 15 μm (tool cutting edge radius of 39 μm). Zhou et al. [13] used the AdvantEdge7.1.002 software to establish a 2D micro turning model, and the model UCT grew gradually with the cutting length, and the MUCT was calculated by the turning point of the cutting force curve, which yielded a MUCT of about 5–6 μm (tool cutting edge radius of 20 μm) for Ti-6Al-4V. Zhi et al. [14] used the Johnson–Cook constitutive model to establish a 2D orthogonal finite element cutting model for PTFE material and discriminated the MUCT by cutting force, machined surface quality, and simulated chip formation, which finally yielded a MUCT of 70 μm (tool cutting edge radius of 40 μm) for PTFE.

Researchers have also conducted experimental studies to determine MUCT. Rahman et al. [15], in their study of cutting Al 6028 aluminum alloy using cubic boron nitride tools, applied surface roughness *Ra* as a criterion for MUCT, and when *Ra* was the lowest, a MUCT of 0.08*r_n_* was obtained. It was found that when the UCT was lower than the MUCT, incomplete and irregularly shaped chips were obtained. Rezaei et al. [16] used a nonlinear increase in specific cutting forces to determine MUCT when performing Ti-6Al-4V dry and minimum quantity lubrication (MQL) cutting and found that the cooling environment directly affects MUCT, which ranged from 0.25 to 0.49*r_n_* for dry cutting, and from 0.15 to 0.34*r_n_* for MQL conditions. Camara et al. [17] utilized the acoustic emission analysis of the MUCT of pure nickel, electrolytic copper, and Al 6262-T6 aluminum alloy; the acoustic emission signal is weakest when the UCT is equal to the MUCT, and the effect of acoustic emission is enhanced when the UCT is less than and greater than the MUCT.

**Figure 1:**
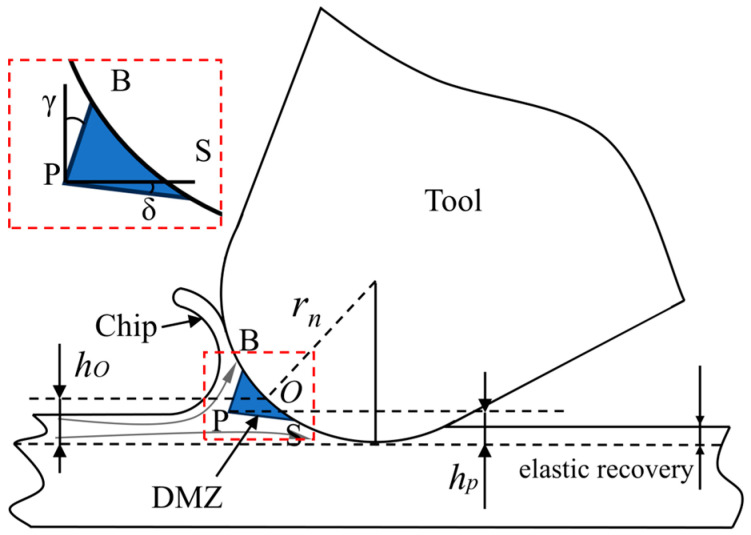
Schematic diagram of micro-cutting DMZ.

In the study of the micro turning process, some researchers [18,19,20] have observed the existence of a triangular region with a very small material flow rate at the rounded corner of the cutting edge of a tool, called the dead metal zone (DMZ). The DMZ forms at the rounded corner of the cutting edge of a tool and acts as a new cutting edge to participate in cutting [21], as shown in Figure 1. The DMZ leads to the appearance of multiple material segregation points in the material instead of just one. Due to the existence of this DMZ, there are at least four separation points: the three vertices of the DMZ, S, B, and P, and the material stagnation point *O*. Several researchers have carried out theoretical modeling and action mechanism studies on the DMZ. Wan et al. [22] combined the plastic molding theory with the slip line field theory to theoretically compute the shape of the DMZ, and for the first time, the positions of the three separation points S, B, and P of the DMZ were computed analytically and deduced, and the MUCT was derived. To simulate the role of DMZ in the micro turning process, Afsharhanaei et al. [23] proposed to artificially introduce the DMZ on the rounded corners of the cutting edge of the tool by altering the tool geometry and simulated this for different rake angle γ and relief angle δ as shown in Figure 1. They found that the transformation of the DMZ to the tool geometry could effectively improve the simulation performance, and the simulation cutting force relative error can be reduced to less than 5%. Hu et al. [24] proposed a new material separation model based on the DMZ, and identified three characteristic points and one stagnation point of the DMZ. Sha et al. [25] proposed a cutting force prediction model based on the DMZ, and set up a finite element model to simulate the distribution of the material flow velocity in the DMZ so as to accurately obtain the position of the material stagnation point *O*.

It can be found that in previous studies on the determination of MUCT, many researchers [6,7,8,12,25] only focus on the determination of MUCT using the stagnation point *O*. In the study of DMZ, some results [22,24] show that stable chips are still generated when the UCT is smaller than the MUCT determined by the stagnation point *O*. The DMZ plays an important role as a material diverter during the cutting process, and its boundaries can constitute a slip line [26], and the material passing through the DMZ will be shunt for two parts: separation point P above the material will flow along the PB up to form chips, and separation point P below the material will flow along the PS down to the bottom of the tool corner, as shown in Figure 1. Therefore, a new MUCT can be determined by the separation point P. Moreover, most researchers [7,10,11,12] only studied the MUCT under one tool cutting edge radius and one cutting parameter, neglecting the effect of cutting conditions on the MUCT.

Based on the above analysis, this paper proposes a new method for determining MUCT using the metal dead zone (DMZ). Micro turning experiments were then conducted to verify the accuracy of the DMZ-based MUCT determination method. Finally, numerical simulations were performed to study the effects of different cutting parameters on DMZ and MUCT. The proposed method, based on finite element simulation, significantly reduces experimental time and cost compared to traditional methods. It also provides a more accurate MUCT than the existing simulation methods, offering new insights for studying the minimum cutting thickness in micro-cutting.

## 2. Numerical Simulations

### 2.1. Numerical Simulation Condition Setting

**Figure 2:**
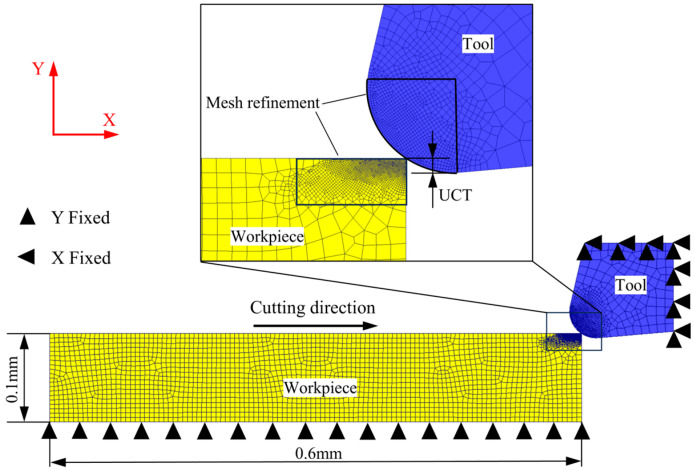
Micro turning finite element simulation model.

An orthogonal micro turning model was established using the DEFORM finite element software. As shown in Figure 2, the rake angle of the tool is 13° and the relief angle is 5°, and the radius of the cutting edge is 40 μm. The workpiece is modeled as a rectangular block with the size of 0.1 mm × 0.6 mm. The tool is completely fixed, the workpiece moves in the X-positive direction, and the movement in the Y-direction is fixed. The tool was set as a rigid body, without considering tool deformation; only the heat transfer factor was considered, and the workpiece was set as a plastic deformed body. The room temperature was set to 20 °C and was applied to the tool and workpiece as the initial temperature condition.

A four-node thermally coupled quadrilateral element was used for mesh division. In the finite element simulation of micro-cutting, the material undergoes significant plastic strain, and conventional Lagrangian methods often lead to severe mesh distortion, causing large computational errors or failure to converge. DEFORM adopts an adaptive Lagrangian mesh delineation method, which is capable of updating the mesh in real time so as to avoid excessive deformation of the mesh. Mesh refinement was applied to the cutting edge of the tool and the cutting deformation zone of the workpiece, with the refined mesh region remaining stationary relative to the tool. The element size of the workpiece mesh refinement area is 0.0003 mm × 0.0003 mm, and the mesh size of the rest of the area is about 0.005 mm × 0.005 mm. The element size of the tool mesh refinement area is about 0.0008 mm × 0.0008 mm, and the mesh size of the rest of the area is about 0.01 mm × 0.01 mm.

### 2.2. Material Properties and Intrinsic Modeling

Titanium alloy Ti-6Al-4V is a typical temperature-sensitive material which generates a large amount of cutting heat during the cutting process, and the material undergoes intense plastic deformation during micro-cutting, so in order to simulate the material behavior under high temperature, large deformation, and high strain conditions, the Johnson–Cook constitutive model is used.

(1) $$\sigma =(A+B\varepsilon^{n})(1+C\ln\frac{\dot{\varepsilon }}{{\dot{\varepsilon }}_{0}})\left[1-{\left(\frac{T-T_{r}}{T_{m}-T_{r}}\right)}^{m}\right]$$

where σ is the equivalent flow stress, ε is the equivalent plastic strain, $\dot{\varepsilon }$ is the equivalent plastic strain rate, ${\dot{\varepsilon }}_{0}$ is the reference strain rate, and *T*, *T_m_*, and *T_r_* are the actual material temperature, melting temperature, and room temperature, respectively. *A*, *B*, *C*, *n*, and *m* are the material parameters. *A* is the yield stress of the material, *B* and *n* denote the effect of strain, *C* is the strain rate constant, and m represents the thermal softening effect. 

The J-C damage model is adopted in this study, and the expression for the damage model is as follows:

(2) $$D=\sum \frac{\Delta \varepsilon }{\varepsilon^{f}}$$

where Δ*ε* is the increment of equivalent plastic strain occurring during an integration cycle, and *ε^f^* is the equivalent strain of fracture.

(3) $$\varepsilon^{f}=\left[d_{1}+d_{2}\exp\left(d_{3}\frac{\sigma_{m}}{\bar{\sigma }}\right)\right](1+d_{4}\ln\frac{\dot{\varepsilon }}{{\dot{\varepsilon }}_{0}})\left[1-d_{5}\left(\frac{T-T_{r}}{T_{m}-T_{r}}\right)\right]$$

where *σ_m_* is the average of the three normal stresses, and $\bar{\sigma }$ is the von Mises equivalent of stress. Table 1 lists the Johnson–Cook constitutive model and damage model parameters for Ti-6Al-4V. The thermophysical properties and basic physical properties of Ti-6Al-4V are given in Table 2 and Table 3, respectively.

### 2.3. Tool–Workpiece Friction Model

**Figure 3:**
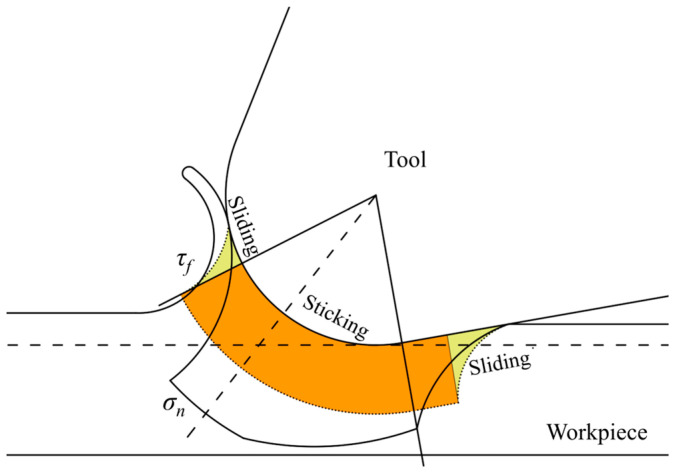
Distribution of normal and shear stresses on the front tool face.

The friction between the tool and the workpiece material is a crucial parameter influencing the MUCT in micro-cutting and affects the generation of frictional heat during the cutting process. Based on the chip formation process in machining, Zorev [30] divided the interaction area between the tool and chip into a sticking region and a sliding region. In micro turning, the locations of the sticking and sliding regions differ from those in conventional cutting; Moon et al. [31] determined the distribution of the bonding and slip zones in the tool corner region based on the material’s plastic flow velocity and the distribution of shear contact forces. This study establishes a cutting friction model based on these two regions to better reflect the friction characteristics in micro-cutting, as illustrated in Figure 3.

In the figure, *σ_n_* denotes the normal stress, and *τ_f_* denotes the friction stress. It can be observed that when the uncut chip thickness (UCT) is small, the chip does not make contact with the rake face of the tool; instead, friction occurs only between the tool’s rounded cutting edge and a portion of the flank face in contact with the workpiece material. As the UCT increases, the extent of the sticking region gradually expands toward the rake face, eventually forming a friction model consistent with that of conventional cutting. The expressions for friction stress in the two friction regions are as follows:

(4) $$\left\{\begin{array}{l} \tau_{f}=\mu \sigma_{n}(\mu \sigma_{n}<{\bar{\tau }}_{\max}),\;\;\;\;\;\;\;\;\;\;\;\;\mathrm{Sliding}\\ \tau_{f}={\bar{\tau }}_{\max}=mK(\mu \sigma_{n}<{\bar{\tau }}_{\max}),\mathrm{Sticking} \end{array}\right.$$

where *τ_f_* is the friction stress, *σ_n_* is the normal stress, *μ* is the friction coefficient, *m* is the shear friction coefficient, *K* is the shear yield stress, and ${\bar{\tau }}_{max}$ is the shear stress. The DEFORM software can judge the type of friction in the cutting process by itself, and simulate the calculation by using the corresponding expression. The tool used in this experiment is a carbide tool, the workpiece material is Ti-6Al-4V, the friction coefficient is set to 0.3 [32], and the shear friction coefficient is the default value of 0.6.

### 2.4. DMZ and MUCT Analysis

**Figure 4:**
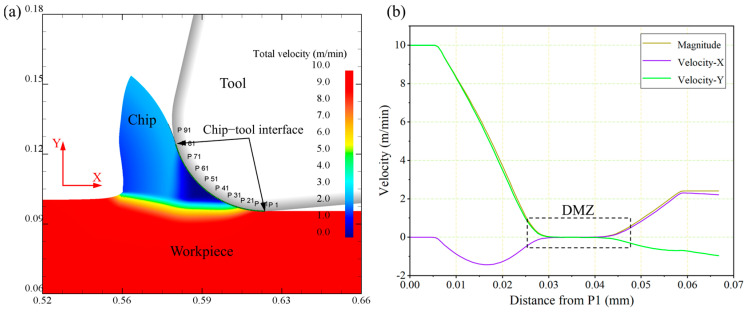
Micro turning DMZ analysis: (**a**) workpiece material flow velocity; (**b**) material motion velocity at the tool–chip contact surface.

An orthogonal cutting simulation experiment was conducted with a cutting speed of 10 m/min and a UCT of 5 μm to observe the material separation behavior. The velocity contour plot of the workpiece material is shown in Figure 4a. It can be observed that chips are generated during the cutting process, and there exists a distinct velocity gradient layer between the chips and the workpiece substrate, which is identified as the shear band. The software automatically identifies the tool–chip contact area, and it is found that the friction region aligns well with the friction model established in the previous section. It can also be seen that there is a triangular dark blue DMZ near the tool’s rounded edge. To further understand the material’s motion during chip formation, 100 points in the tool–chip contact region were uniformly sampled to extract their velocity, which is plotted and analyzed as shown in Figure 4b. It is evident that all three velocity curves exhibit a phase where the material velocity is relatively low, with values close to but not exactly zero, corresponding to the flow characteristics within the DMZ. To standardize the definition of material flow inside the DMZ and ensure consistency with the simulation analysis, the region where the resultant flow velocity is less than 10% of the cutting speed is regarded as the DMZ.

**Figure 5:**
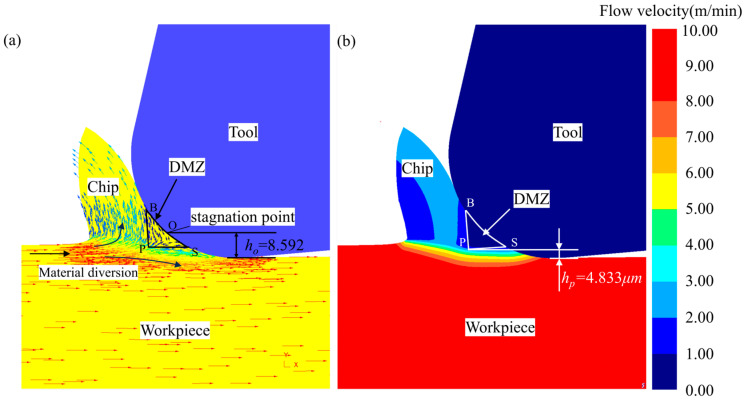
Two types of MUCT measurement: (**a**) flow velocity vector; (**b**) flow velocity contour.

Figure 5a shows the nodal velocity of the material during the micro-cutting process, which can be used to illustrate the flow of the material, and the length of the line segment of the arrow represents the magnitude of the motion velocity. It can be seen that the workpiece material diverges close to the tool edge fillet, and a part of the material moves at a reduced velocity and flows upwards to form chips; a part of the material flows downwards along the tool edge radius to form the machined surface. According to the MUCT prediction formula based on stagnation point O proposed by Son et al. [7], we calculated that the MUCT is 7.909 μm, measuring the simulation results of the stagnation point *O* to the bottom of the tool distance to obtain the MUCT based on the stagnation point *O*, *h_O_* = 8.592 μm. The simulation UCT of 5 μm is obviously smaller than the *h_O_*, and from Figure 5a, we obviously see that the cutting process has a stable chip generation, so the accuracy of the MUCT of Ti-6Al-4V determined by the stagnation point O is relatively low. 

Figure 5b shows the contour cloud diagram of material flow velocity. Based on the DMZ definition, where the material flow velocity is less than 10% of the cutting speed, the DMZ can be approximated as a triangular-shaped SBP. At the apex P of the triangular DMZ, there is a noticeable material flow bifurcation. The distance from point P to the bottom of the tool, measured as MUCT *h_p_*, is 4.833 μm, which corresponds to approximately 12.08% of the tool cutting edge radius.

## 3. Experiment Validation

### 3.1. Experimental Setup

**Figure 6:**
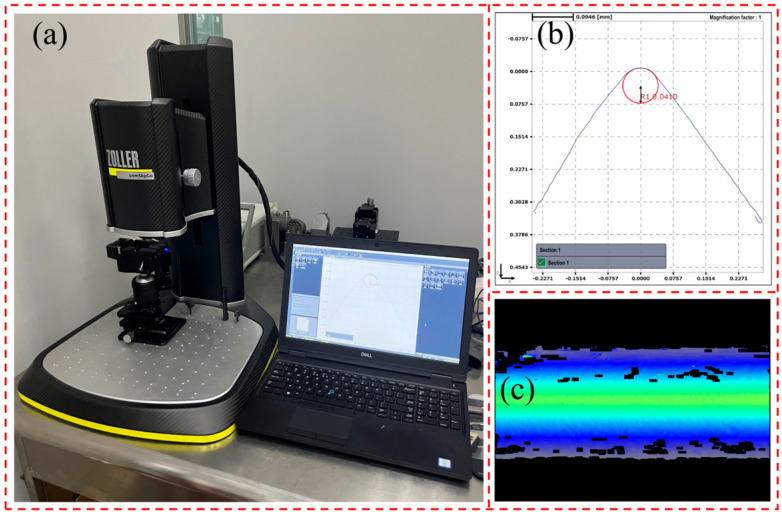
Tool presetting gauge to measure the radius of the cutting edge of tool: (**a**) tool presetting gauge; (**b**) cutting edge profile line; (**c**) cutting edge profile.

The experimental workpiece is a Ti-6Al-4V titanium alloy hollow tube, with an outer diameter of 16 mm, wall thickness of 3 mm, and the length of 50 mm. The cutting tool used is a VBMT160404-MV (Taizhou, China) coated carbide insert, featuring a TiCN coating applied via PVD, providing excellent hardness and wear resistance. The tool’s rake angle is 13°, and the relief angle is 5°. The tool holder model is SVJBR2020K16 (Taizhou, China). As shown in Figure 6, the radius of the main cutting edge of the tool was measured using the ZOLLER pomSkpGo tool presetting gauge (Michigan, America). After taking three measurements and averaging the results, the tool cutting edge radius was determined to be approximately 40 μm.

**Figure 7:**
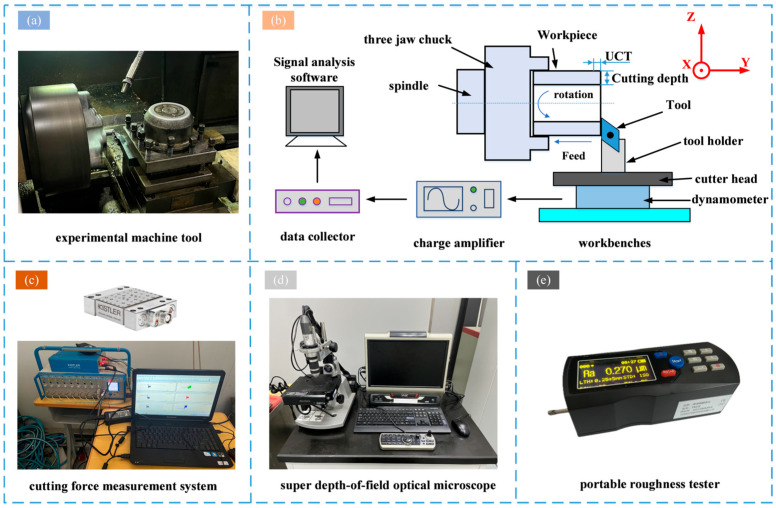
Experiment system and experimental equipment: (**a**) experimental machine tool; (**b**) cutting experiment procedure; (**c**) cutting force measurement system; (**d**) super depth-of-field optical microscope; (**e**) portable roughness tester.

The orthogonal experiment was conducted on a CAK4085NJ CNC lathe produced by Shenyang Machine Tool Works (Shenyang, China), with a maximum spindle power of 7.5 kW and a maximum speed of 2000 rpm. The cutting force measurement system consisted of a Kistler9257B cutting force plate produced by Kistler Instruments, a Kistler5070 charge amplifier (Hampshire, UK), a data collector, and a computer with the DynoWare signal processing software (Tape 2825A, Winterthur, Switzerland). The DynoWare signal processing software was used to collect the cutting force in both X- and Y-directions during the experiment. The experiment setup is shown in Figure 7. The experiment was conducted using orthogonal free cutting, and before the experiment, the tool holder was adjusted so that the main cutting edge of the insert was perpendicular to the feed direction, and the length of the main cutting edge of the insert was 16 mm, which was greater than the wall thickness of the workpiece by 3 mm, to ensure that only the main cutting edge was involved in the cutting. After the experiment, a VMX-2000C (Shenzheng, China) super depth-of-field optical microscope was used to analyze the image of the chips and the machined surface of the workpiece, and a TR221 portable roughness tester (Beijing, China) was used to check the surface roughness. A new tool was used for each experiment to ensure data accuracy. The design of the UCT experimental process parameters is shown in Table 4.

### 3.2. Comparative Analysis of Cutting Forces

**Figure 8:**
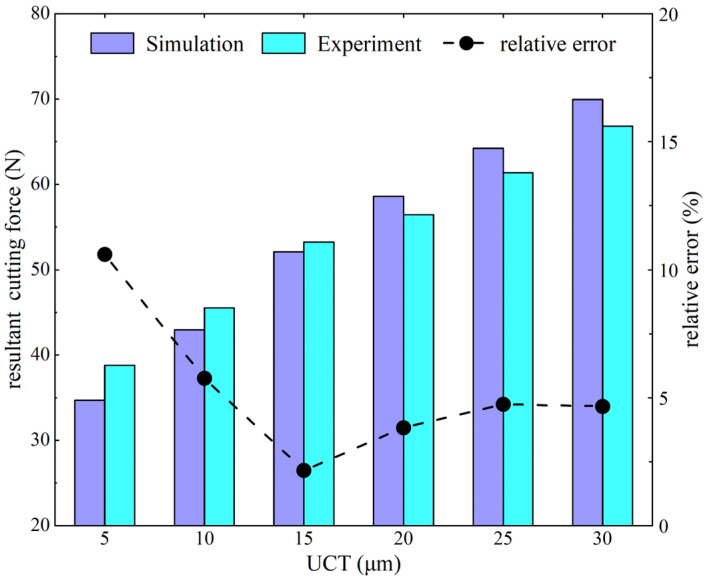
Comparison of simulated and experimented resultant cutting forces.

Cutting simulation experiments were performed using the established 2D simulation model with a cutting speed of 10 m/min, a cutting depth of 3 mm, and a UCT of 5 μm, 10 μm, 15 μm, 20 μm, 25 μm, and 30 μm. The average resultant cutting forces in the cutting direction obtained from the simulation and experiment are shown in Figure 8. It can be observed that the cutting force increases with an increase in UCT. The maximum relative error between the simulation and experiment results was 10.59% for a UCT of 5 μm. The minimum relative error between simulation and experiment was 2.16% for a UCT of 15 μm. The error can be attributed to the fact that the simulation was carried out in an idealized situation, and the cutting force in the actual cutting process will be affected by cutting vibration, machine vibration, and clamping and other factors [33]; on the whole, the simulation results are more in line with the experiment, and the established cutting simulation model has a high level of accuracy.

### 3.3. Chip-Based MUCT Analysis

**Figure 9:**
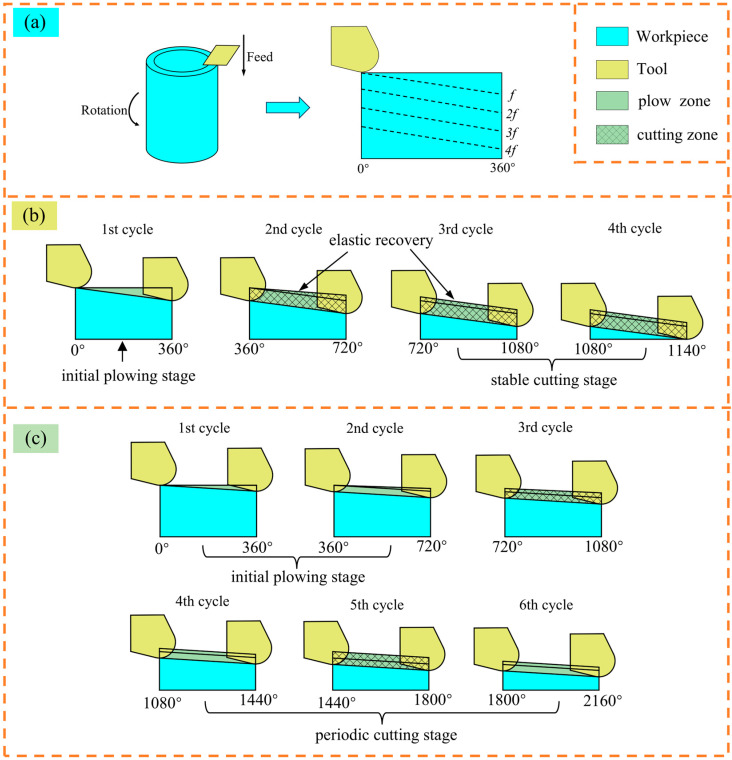
Chip formation process. (**a**) Simplified cutting process; (**b**) UCT equal to MUCT cutting process; (**c**) UCT lower than MUCT cutting process.

In the micro turning process, chips are generated at feeds greater or less than MUCT, and complete strips of chips are generated continuously at feeds greater than MUCT, and incomplete, shorter, and irregular chips are generated intermittently at feeds less than MUCT so that the MUCT can be analyzed by the morphology of the chips [15]. The chip generation process of this experimental scheme can be simplified as a multi-cycle cutting process, as shown in Figure 9a. When UCT is equal to MUCT, it enters the stable cutting stage after one cycle of the plowing stage, forming complete strip chips, as shown in Figure 9b; when UCT is smaller than MUCT, it enters the periodic cutting stage after a certain cycle of the plowing stage, and the cutting process will be cyclic in plowing cutting and shearing cutting, forming incomplete or irregular chips, as shown in Figure 9c.

**Figure 10:**
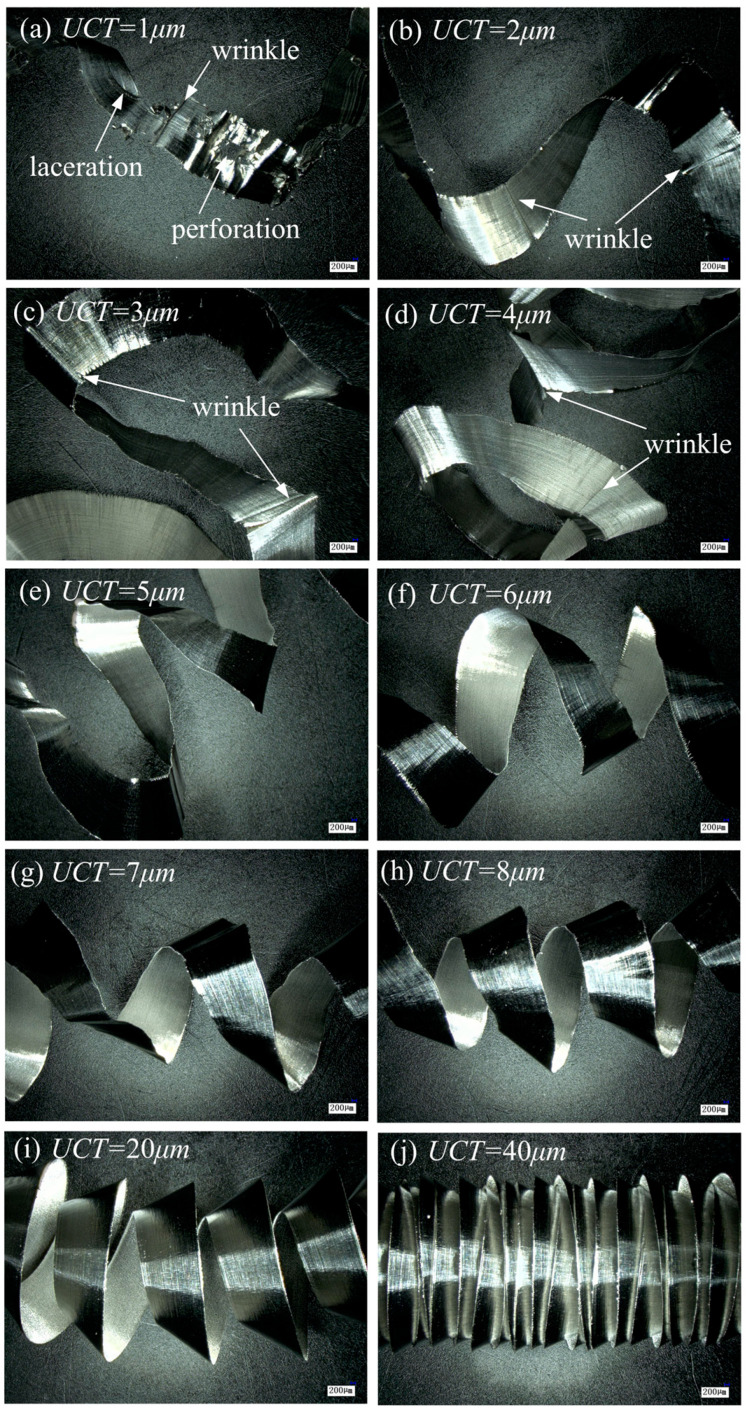
Chip morphology for different UCTs.

Figure 10 shows the microscopic images of the chips produced at different UCTs. It can be found that the shape of the chip produced at a UCT of 1 μm is irregular and there are more perforations, wrinkles, and lacerations on the surface, as shown in Figure 10a. The perforations and lacerations are caused by the insufficient material removed by the tool and the low stiffness of the chips that cannot withstand the deformation of the material, and the wrinkled area is an irregular chip formed by a small number of chips generated by the plow cutting. As can be seen from Figure 10b–d, the chips generated when the UCT is 2–4 μm are irregular, the surface appears obvious wrinkles, and stable cutting is not achieved. When the UCT is 5 μm, the chips are complete and show a regular spiral shape, as shown in Figure 10e. Therefore, the MUCT of Ti-6Al-4V is about 5 μm, aligning closely with the simulation results for MUCT, and the relative error is about 3.34%, indicating the accuracy of determining MUCT using the DMZ vertex P method.

From Figure 10a–j, it can be found that with the increase in UCT, the curl radius of the chip decreases gradually, and the pitch of the chip helix decreases gradually. This is because, during cutting, a significant amount of cutting heat is transferred to the chips, with the highest temperature at the tool–chip interface. The chip thickness increases with a higher UCT, creating a higher temperature gradient across the chip’s surfaces. Consequently, the material on the chip side in contact with the tool undergoes greater expansion than the opposite side, resulting in chips with higher curvature [33].

### 3.4. MUCT Analysis Based on Surface Quality

**Figure 11:**
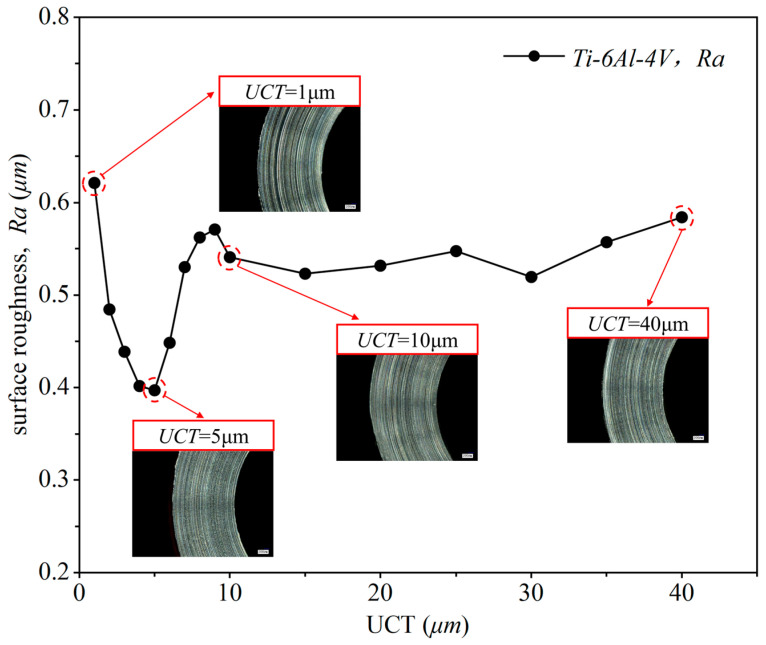
Effect of UCT on surface quality.

The researchers [15,33] also used the surface quality of the machined workpiece to evaluate the MUCT. They found that when the UCT equals the MUCT, the surface roughness reaches a local minimum. Therefore, this study utilizes surface quality to analyze the MUCT and further validate the simulation results. The effect of different UCTs on the surface quality of the machined surface is shown in Figure 11. As illustrated, when the UCT is low, surface roughness increases as UCT decreases. This differs from the results observed in traditional cutting processes. The reason for this is that when UCT is below MUCT, only a small amount of material is removed in cycle 1. In the next cycle, UCT accumulates until it reaches MUCT, at which point a large amount of chips starts to form, as seen in cycles 1–3 in Figure 9c. As UCT decreases, the cutting process involves multiple cycles 2 until the accumulated UCT reaches MUCT. This makes the initial plowing phase longer, and increases the number of plowing cycles in the periodic cutting stage (cycles 4–6 in Figure 9c). The plowing effect creates scratches on the workpiece surface, which reduces surface quality [34]. Therefore, as UCT decreases, more plowing cycles occur, leading to more scratches and poorer surface quality. 

At a UCT of 5 μm/r, the surface roughness reaches its minimum value, and the surface quality of the workpiece improves, with almost no deep scratches. This is because when UCT equals MUCT, the cutting process only goes through the plowing phase during the 1st cycle (as shown in Figure 9b). In the stable cutting phase, stable, complete, and regular chips are formed, and the shear force gradually becomes dominant, significantly reducing surface scratches. Additionally, the tool compresses some of the material peaks on the rough surface into the valleys, further reducing surface roughness. As the UCT continues to increase beyond 5 μm, the cutting process is similar to Figure 9b, but in this case, UCT reaches MUCT during the 1st cycle, forming stable chips and entering the stable cutting phase. But with the cutting force rises, leading to greater plastic deformation of the material and increased machine vibrations, which deteriorate the surface quality. Therefore, the MUCT derived from the surface quality analysis is about 5 μm, consistent with the MUCT obtained through the DMZ method, demonstrating the high accuracy of this calculation approach.

## 4. Results and Discussion

### 4.1. Effect of UCT on Equivalent Stress and Equivalent Strain Rate

**Figure 12:**
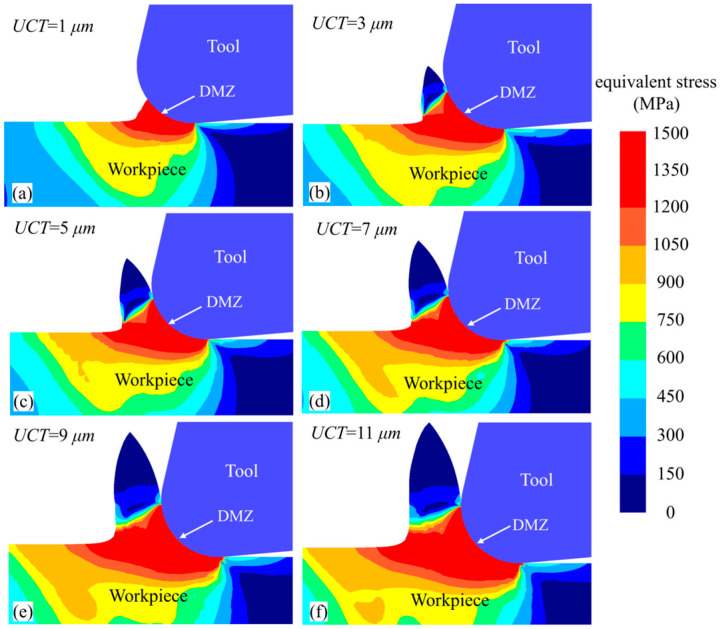
Equivalent stress cloud of different UCT workpieces at a cutting length of 0.2 mm.

Using the established 2D simulation model, cutting simulation experiments were conducted at a cutting speed of 10 m/min, a cutting depth of 3 mm, and UCTs of 1 μm, 3 μm, 5 μm, 7 μm, 9 μm, and 11 μm. The equivalent strain distribution in the deformation zone is shown in Figure 12. Under the effect of the DMZ, a “y”-shaped red high-stress band forms within the deformation zone. As the UCT increases, the area of this high-stress region also expands, indicating that more workpiece material undergoes intense plastic deformation. This is because an increase in UCT diminishes the negative rake effect of the tool’s rounded edge, thereby strengthening the shearing action and allowing more material to undergo shear deformation to form chips. Moreover, it can be observed that when the UCT is less than the MUCT (minimum uncut chip thickness), minimal chip formation occurs as material accumulates at the tool’s rounded edge, as illustrated in Figure 12a,b, which is consistent with the results obtained from the cutting experiment.

**Figure 13:**
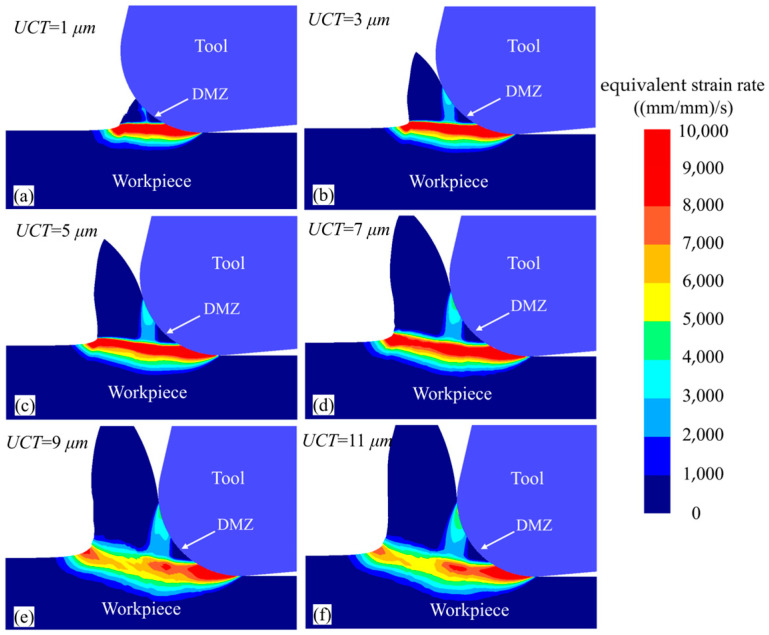
Equivalent strain rate cloud of different UCT workpieces at a cutting length of 0.2 mm.

The equivalent strain rate distribution in the deformation zone is shown in Figure 13. Under the influence of DMZ, a similar “y”-shaped high equivalent strain rate band forms in the deformation zone. The boundary of the DMZ can be considered the boundary of the micro-cutting plastic deformation zone, aligning well with the findings of Waldorf et al. [35]. As the UCT increases, the equivalent strain rate band expands in length and area, while the equivalent strain rate gradually decreases. This trend occurs because at lower UCT values, the negative rake effect of the tool’s rounded edge is stronger, leading to a minimal shear angle and rapid material flow in the workpiece, which generates a high equivalent strain rate. In contrast, at larger UCT, the rounded edge exhibits a smaller negative rake angle and a larger shear angle, allowing easier shear deformation of the material, resulting in a lower equivalent strain rate in the shear band. Due to the small UCT, the DMZ is not fully formed in Figure 13a,b, resulting in a relatively small DMZ area. From Figure 13c–f, it can be seen that as the UCT increases, the size and position of the DMZ remain nearly constant, indicating that UCT has minimal effect on the DMZ, which is consistent with the characteristics of MUCT [36].

### 4.2. Effect of Tool Cutting Edge Radius on MUCT

**Figure 14:**
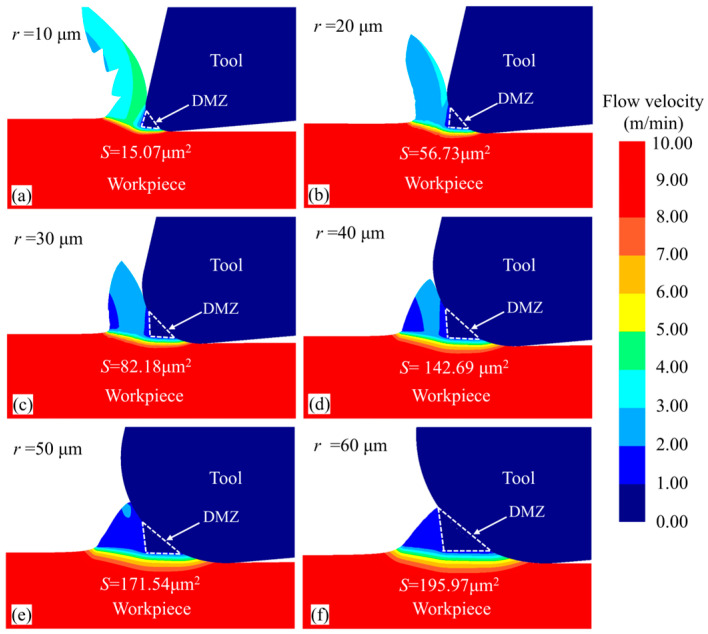
Material flow rate cloud of workpieces with different cutting edge radii at 2000 steps.

**Figure 15:**
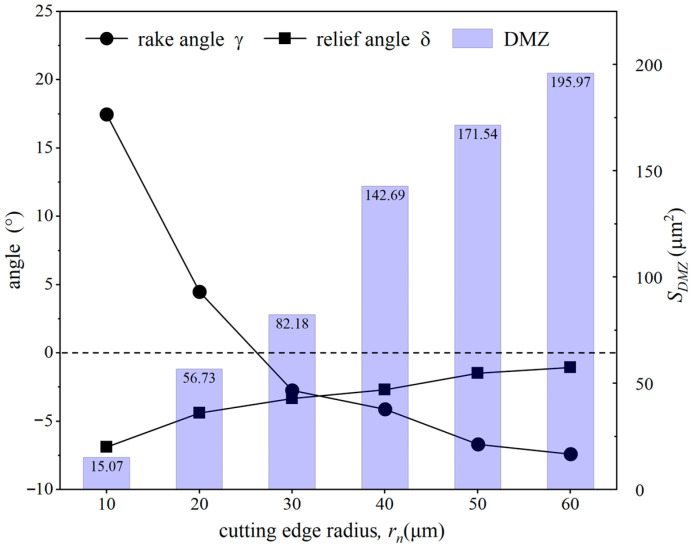
Effect of tool edge radius on the rake angle, relief angle, and area of the DMZ.

Figure 14 shows the material flow velocity contour for the workpiece under different tool cutting edge radii at the same simulation steps. Observing the chip morphology in front of the tool’s rounded edge, it can be seen that as the tool edge radius increases, the chip thickness gradually increases, but the chip volume decreases and eventually nearly no chips are generated. Additionally, as the tool edge radius increases, the geometry of the DMZ changes significantly. The rake angle (γ), relief angle (δ), and area of the DMZ were measured, with the results shown in Figure 15. It can be observed that with increasing tool edge radius, the relief angle of the DMZ gradually increases, while the rake angle (γ) gradually decreases, eventually becoming a negative rake angle. This makes it more difficult for the material to flow upward to form chips, resulting in more material accumulation in front of the rounded edge. The accumulated material faces greater difficulty in bypassing the DMZ to form chips; consequently, more material flows into the DMZ and becomes part of it, leading to a rapid increase in the DMZ area.

**Figure 16:**
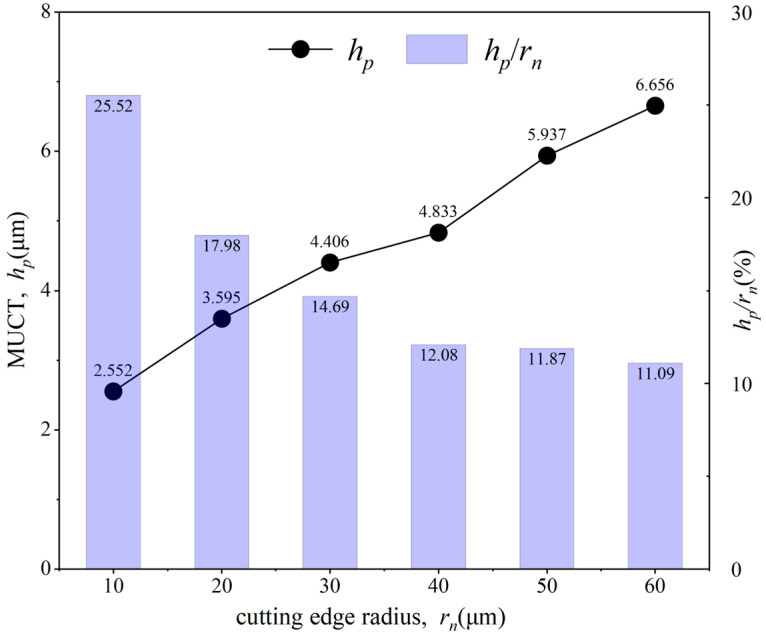
Effect of different cutting edge radii on MUCT.

The minimum uncut chip thickness (MUCT, *h_p_*) was measured and calculated for each tool cutting edge radius, and the ratio of MUCT to the tool cutting edge radius (*h_p_*/*r_n_*) was also computed, with the results shown in Figure 16. It can be observed that the MUCT increases linearly with the tool cutting edge radius. For the same material, *h_p_*/*r_n_* is generally considered a fixed value [10,11,36]. However, the experimental results show that *h_p_*/*r_n_* gradually decreases with increasing tool cutting edge radius, and the rate of decrease becomes slower. The reason for this is analyzed as follows: with an increasing tool cutting edge radius, the actual negative rake angle at the contact area between the rounded edge and the material does not increase linearly. Instead, as the radius increases, the actual rake angle reduction in the contact zone gradually lessens.

### 4.3. Effect of Cutting Speed on MUCT

**Figure 17:**
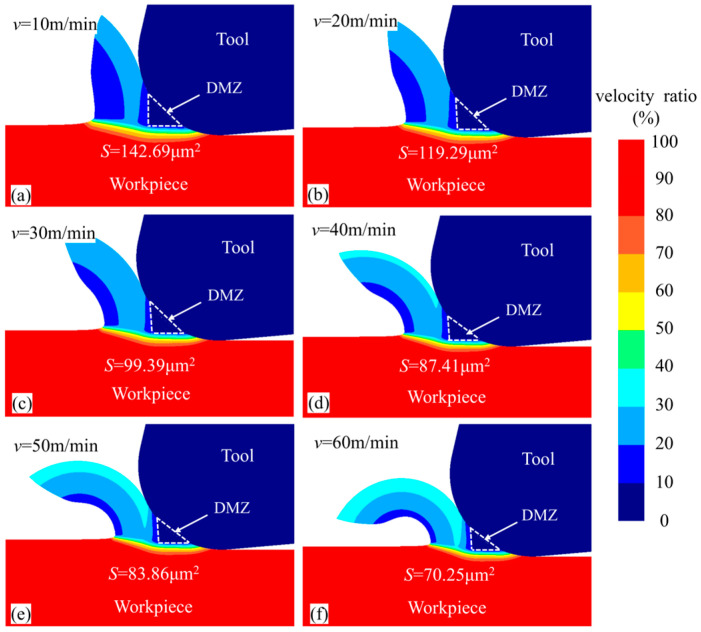
Material flow rate cloud for different cutting speeds at 2000 steps.

**Figure 18:**
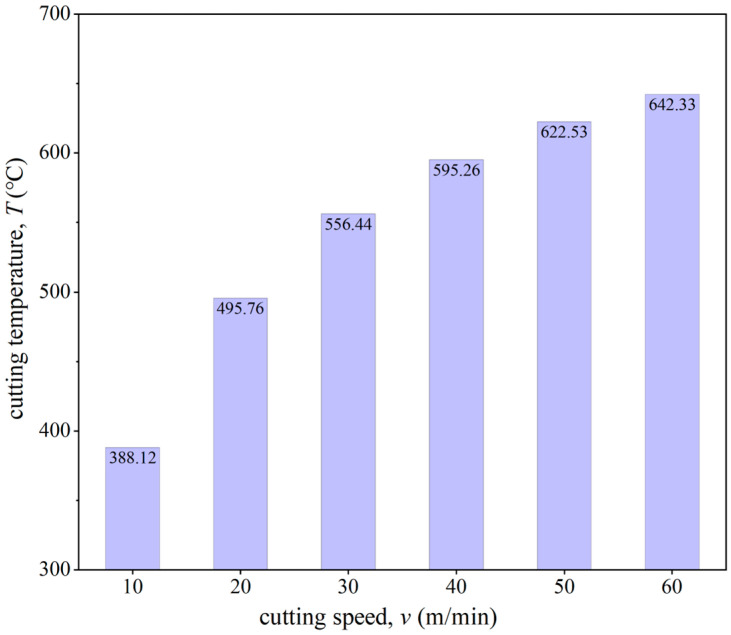
Average cutting temperature in the cutting deformation zone during the cutting process.

Figure 17 presents the material flow velocity contour of the workpiece at different cutting speeds for the same simulation steps. The DMZ area was measured and calculated for each tool cutting edge radius. When the cutting speeds *v* were 10 m/min, 20 m/min, 30 m/min, 40 m/min, 50 m/min, and 60 m/min, the DMZ areas were 142.69 μm^2^, 119.29 μm^2^, 99.39 μm^2^, 87.41 μm^2^, 83.86 μm^2^, and 70.25 μm^2^, respectively. It was observed that with increasing cutting speed, the DMZ area gradually decreased. Figure 18 shows the average cutting temperature in the cutting deformation zone during the cutting process. It can be found that with the increase in cutting speed the cutting temperature in the cutting deformation zone gradually increases, and the increase is gradually reduced. The increase in cutting temperature will have a softening effect on the material, reducing the difficulty of shear deformation so that more material forms chips, resulting in a reduction in the DMZ.

**Figure 19:**
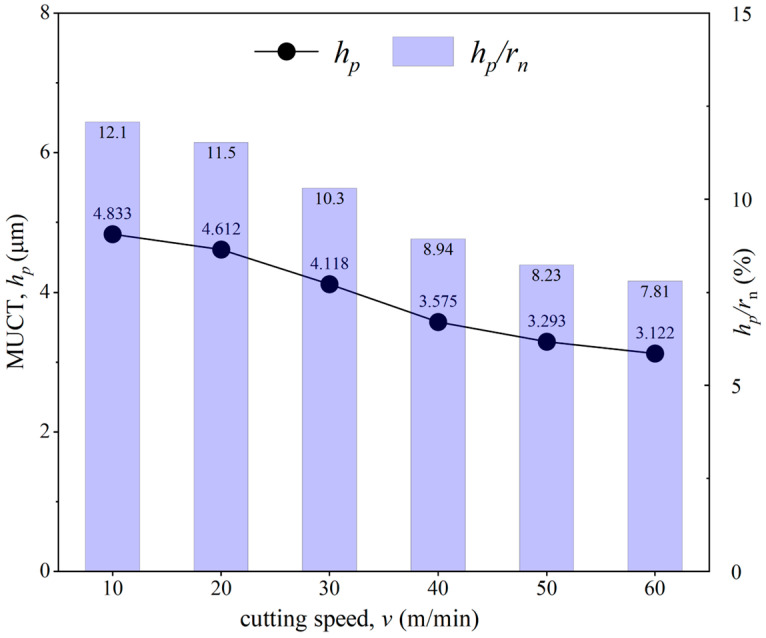
Effect of different cutting speeds on MUCT.

The MUCT was measured and calculated at each cutting speed, and the ratio *h_p_*/*r_n_* was also computed, with the results shown in Figure 19. It can be seen that both MUCT and the ratio *h_p_*/*r_n_* decrease as the cutting speed increases. This indicates that cutting speed influences MUCT: the higher the cutting speed, the higher the temperature in the cutting deformation zone, leading to a smaller MUCT. These findings are consistent with the experimental results by Rezaei et al. [16].

## 5. Conclusions

The paper investigates the effect of DMZ on micro-cutting material shunt by finite element simulation, determines the DMZ range, and proposes a new method to determine the MUCT based on DMZ. In order to verify the simulation model and the accuracy of the obtained MUCT, cutting experiments were conducted to verify the analyzed results. Finally, the effects of different cutting conditions on DMZ and MUCT were investigated. The main conclusions are summarized as follows:

1. The proposed method of determining the MUCT by DMZ is reliable. The simulation results are in good agreement with the experimental results, and the relative error of cutting force ranges from 2.16% to 10.59%, and the relative error of the MUCT is about 3.34%.
2. The equivalent stress and equivalent strain rate at the DMZ boundary are both high, which can be regarded as the boundary of the micro-cutting plastic deformation zone. The UCT only affects the formation speed of the DMZ, with minimal impact on the position and geometry of the DMZ.
3. The tool cutting edge radius significantly influences both the DMZ and MUCT. As the tool cutting edge radius increases, the DMZ area and MUCT increase as well. Furthermore, the *h_p_/r_n_* value varies with the tool’s cutting edge radius rather than remaining constant, decreasing as the cutting edge radius increases.
4. Cutting speed has a certain impact on the MUCT; as the cutting speed increases, the temperature in the cutting deformation zone rises, leading to a decrease in both the MUCT and *h_p_/r_n_*.

## Figures and Tables

**Table 1:** Johnson–Cook constitutive model and damage model parameters for Ti-6Al-4V [27].

J-C	*A* (MPa)	*B* (MPa)	*C*	*n*	*m*
	1098	1092	0.014	0.93	1.1
Damage	*d*_1_	*d*_2_	*d*_3_	*d*_4_	*d*_5_
	−0.09	0.27	−0.5	0.014	3.87

**Table 2:** Thermophysical properties of Ti-6Al-4V [28].

Temperature (K)	300	373	473	573	673	773
Thermal conductivity (w/(m⸱K))	6.8	7.4	8.7	9.8	10.3	11.8
Specific heat (J/(Kg/K))	611	624	653	674	691	703

**Table 3:** Basic physical properties of Ti-6Al-4V [29].

Density *ρ* (kg/m^3^)	Modulus of Elasticity *E* (GPa)	Poisson’s Ratio *μ*	Coefficient of Linear Expansion (10^−6^ K^−1^)	Inelastic Thermal Coefficient
4430	110	0.33	9	0.9

**Table 4:** Experiment parameters.

Cutting Parameters	Digital
Cutting speed	10 m/min
Spindle speed	200 r/min
Cutting depth	3 mm
Uncut chip thickness (UCT)	1–40 μm

## Data Availability

The original contributions presented in the study are included in the article; further inquiries can be directed to the corresponding author.
